# A study on the use of the Osstell apparatus to evaluate pedicle screw stability: An in-vitro study using micro-CT

**DOI:** 10.1371/journal.pone.0199362

**Published:** 2018-06-28

**Authors:** Daisuke Nakashima, Ken Ishii, Morio Matsumoto, Masaya Nakamura, Takeo Nagura

**Affiliations:** 1 Department of Orthopedic Surgery, Keio University School of Medicine, Shinjuku, Tokyo, Japan; 2 Department of Orthopedic Surgery, International University of Health and Welfare School of Medicine, Narita, Chiba, Japan; 3 Department of Clinical Biomechanics, Keio University School of Medicine, Shinjuku, Tokyo, Japan; Rush University Medical Center, UNITED STATES

## Abstract

Pull-out force and insertion torque have not been generally used as intraoperative measures for the evaluation of pedicle screw stability because of their invasiveness. On the other hand, resonance frequency analysis is a non-invasive and repeatable technique that has been clinically used in dentistry to evaluate implant stability e.g. by the Osstell apparatus. In this study, the characteristics of the implant stability quotient (ISQ) value obtained by the Osstell apparatus in the field of spinal surgery were investigated. Biomechanical test materials simulating human bone were used to provide a comparative platform for evaluating each fixation strength measure, including pull-out force, insertion torque, and the ISQ value. To perform pull-out force measurement and to repeat pedicle screw insertion and removal, loosening was artificially created, and its effect was investigated. The grade of loosening was quantified on a micro-CT image after pedicle screw removal. In the comparison of the 3 fixation strength measures, the correlations of the ISQ value with the pull-out force (R^2^ = 0.339 p <0.0001) and the insertion torque (R^2^ = 0.337 p <0.0001) were lower than the correlation between pull-out force and insertion torque (R^2^ = 0.918 p <0.0001). On a micro-CT study, the material volume of the internal threads disappeared after destruction of its integrity due to repeated pedicle screw insertion and removal. Material integrity destruction of the internal threads decreased only the pull-out force and the insertion torque, but it did not affect the ISQ value. The ISQ value only decreased when the material volume of the internal threads disappeared, probably because the ISQ value reflects the resistance against a force in the perpendicular direction of the screw, unlike the conventional measures of fixation strength, such as pull-out force and insertion torque, which reflect axial load.

## Introduction

Implant stability is important in the field of orthopedics, because it secures implant fixation to the bone and is needed to avoid implant fixation failure [1–4]. Especially in the field of spinal surgery, the rates of postoperative pedicle screw loosening were reported to be as high as 12% [5–7]. However, there is no general method to intraoperatively assess the initial fixation strength of pedicle screws.

Pull-out force [1, 8–12] and insertion torque [1, 13, 14] are the measures of fixation strength that are generally used to evaluate the stability of implants, especially screws. Screw fixation strength, also referred to as pull-out force, is measured destructively through laboratory mechanical testing and is defined as the maximum axial force required to pull the screw out from the bone [15]. This fixation strength cannot be measured in clinical situations because of its invasiveness. In contrast, insertion torque can be measured during surgery. However, because insertion torque is measured during screw insertion measurement, it is impossible to measure insertion torque more than once after the screw has been fixated. Due to these reasons, pull-out force and insertion torque are not generally used as intraoperative measurement methods.

Several studies have reported on the noninvasive measurement of orthopedic joint prosthesis stability during and after surgery [16–22]. In particular, the method of using vibration was introduced by Lippmann [23] in 1932 and has been reported by several authors. In the field of hip surgery, the vibration analysis of the femoral stem and acetabular cup during total hip replacement has been reported [18–20, 22]. In the field of knee surgery, Leuridan et al reported the possibility of vibration method to assess the knee implant stability [24]. As a postoperative method, subcutaneous accelerometers can measure vibrations from a device placed on the skin [18]. Despite these numerous reports on hip surgery, there has not been any widely prevalent method in the field of orthopedic surgery and there are no reports on spinal surgery.

In the field of dentistry, resonance frequency analysis (RFA) is performed widely for vibration analysis during surgery [25–27]. The Osstell ISQ® (Osstell; Integration Diagnostics, Göteborg, Sweden) is used to measure the resonance frequency by vibrating the dental implant with a magnetic pulse [26]. This magnetic excitation signal is a sine wave with peak amplitude of 1 V. At the first flexural resonance, there is a marked increase in amplitude and a change in the phase of the received signal. This can be illustrated graphically as a Bode plot of frequency against amplitude [28]. The result of the measurement is presented as a dedicated parameter, which is the implant stability quotient (ISQ), which ranges from 0 (lowest stability) to 100 (highest stability) [28]. An ISQ value of 0 represents a resonance frequency of about 3000 Hz, and an ISQ value of 100 represents a resonance frequency of about 8000 Hz [29]. This non-invasive and repeatable technique could reflect multidirectional fixation strength [28, 30, 31]. In the field of dentistry, the ISQ value was reported to correlate with the percentage of the implant in contact with the bone (i.e., bone-to-implant contact ratio) [32, 33]. Using this property, the ISQ value has been used to measure *in vivo* the temporal changes in implant stability that accompany the changes in bone structure around the implant and provide the diagnosis of implant loosening [28, 30]. Although the relationship of the ISQ value with the conventional measures of fixation strength, such as the insertion torque [34], and with the structure of the tissue surrounding the implant has been studied, there have been no studies in the field of spinal surgery.

The aim of this study was to investigate, for the first time, the characteristics of the ISQ value in the field of spinal surgery, in comparison with the conventional methods of fixation strength measurements, such as insertion torque and pull-out force. In addition, we aimed to examine the relationship of these 3 fixation strength measures with the surrounding bone structure of the implants using micro-CT images by artificially creating implant loosening.

## Materials and methods

### Biomechanical test materials

Five solid rigid polyurethane forms (Catalogue no. #1522-04, Pacific Research Laboratory, Inc., Vashon Island, WA, USA) were prepared as the test objects to represent human vertebrae. This material was constructed from rigid polyurethane foam with a density of 0.32 g/cm^3^. These biomechanical test materials provided a homogeneous and consistent material that was similar to human cancellous bone [35]. These materials conformed to the American Society for Testing and Materials (ASTM) standard and were shaped into 60 mm × 60 mm × 40 mm blocks.

### Pedicle screw insertion

**Fig 1:**
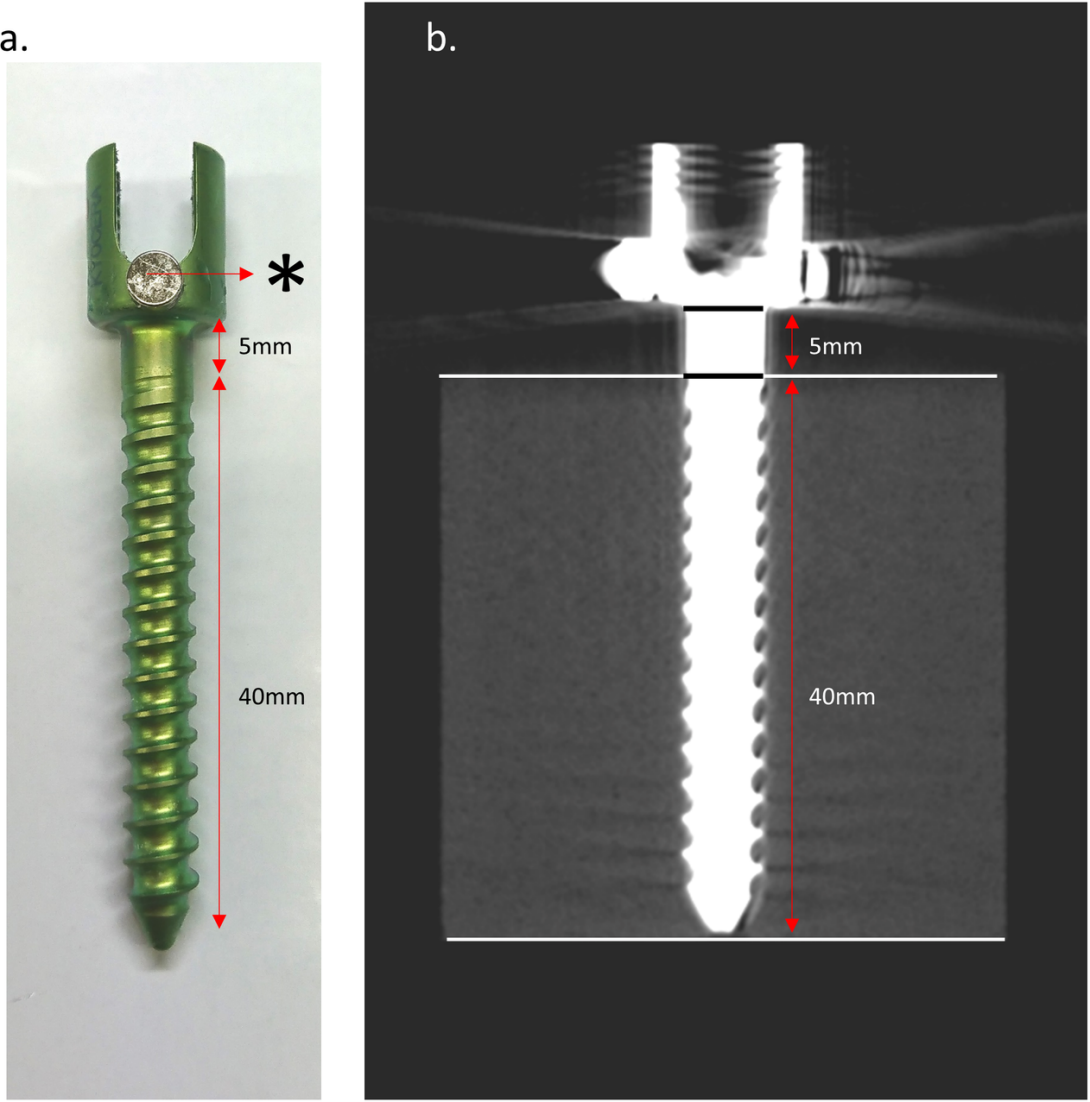
**Photograph of spinal instrumentation with magnets (a) and micro-CT image after screw insertion (b).** Micro-CT confirms that only 40 mm is inserted into the biomechanical test material. *neodymium magnet.

Five single-threaded, non-cannulated, titanium alloy (Ti-6Al-4V (ELI), ASTM F136), monoaxial pedicle screws (Catalog no. CMS05135, Kyocera Medical Corporation, Osaka, Japan) with length of 45 mm, outer threaded diameter of Ø 5.5 mm, inner thread diameter start point of Ø 3.8 mm – end point of Ø 4.6 mm, and screw pitch of 2.5 mm were used. To measure RFA with the Osstell ISQ® (Osstell; Integration Diagnostics, Göteborg, Sweden), 2 neodymium magnets (Magfine, Miyagi, Japan) with a diameter of 4 mm, height of 2 mm, surface inductive flux of 331.7 mT, and magnetic attraction of 0.345 kgf were attached to the head of the pedicle screw using a cyanoacrylate-type of chemically reactive adhesive (Konishi Co., Osaka, Japan). The magnets’ magnetic poles were mounted face to face (Fig 1A).

A posthole was dug at the center of the 60 mm × 60 mm material. Before insertion, a pilot hole was drilled into the material using a 1.5-mm drill bit. A pedicle screw was inserted in its 40-mm length, leaving 5 mm not inserted. All screws were inserted in the same depth (40 mm) using a consistent depth gauge, and micro-CT examinations were performed to check the implanted screw depths (Fig 1B).

### Micro-CT image analysis

**Fig 2:**
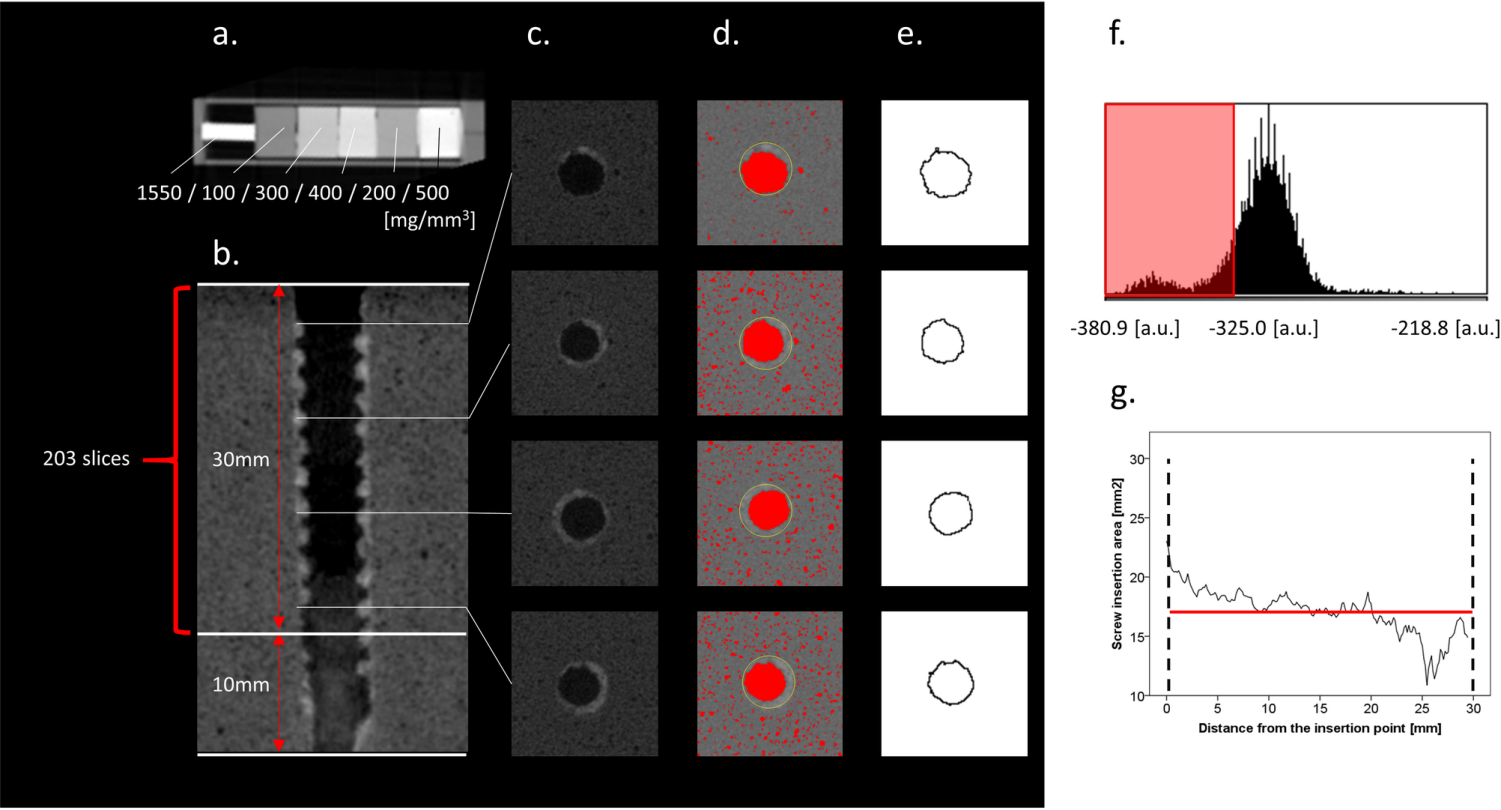
Micro-CT images and image analysis. (a) The bone mineral density phantom made from aluminum alloy and hydroxyapatites. The values indicate the original bone mineral density values. (b) Sagittal image of the screw insertion area after screw removal. (c) Axial images of the screw insertion area. (d) Red areas represent the areas with values lower than −325.0 a.u. (i.e., “air”). The yellow circles represent the 5.5-mm outer diameter of the screw. We defined the location of air surrounded by the yellow circle as the screw insertion area. (e) Actual measurement area. (f) Histogram of the calibrated values. (g) Example of the screw insertion area after first screw insertion and removal. The red line is the average screw insertion area.

The screw-inserted materials were scanned to check the depths of the pedicle screws (Fig 1B) and the shape of screw insertion holes after screw removal or the screw insertion area (Fig 2B and 2C). The conditions of the micro-CT system (R_mCT2 FX, Rigaku Corporation, Tokyo, Japan) were as follows: X-ray voltage 90 kV, current 160 μA, 120 seconds, continuous non-stepping rotation, four frames averaging, rotation over 360°, used a 2.5-mm-thick aluminum filter for reduction of beam hardening, and integration time of 33 ms, saved as 16,384 grey-level images. For the bone mineral density (BMD) phantom (RATOC, Tokyo, Japan) (Fig 2A) and each material, a stack of 400 cross-sectional slices (corresponding to a total height of 57 mm) was reconstructed, with a slice-to-slice distance of 1 pixel (144 μm). Each time before the materials were scanned, the BMD phantom was scanned (Fig 2A). This phantom was originally used in bone research for the conversion from CT number (Hounsfield units) to the BMD value (mg/mm^3^). In the present study, it was used for converting the CT number to the calibrated value (arbitrary unit: a.u.) for quantification.

A total of 203 slices calibrated by the BMD phantom (Fig 2A) were obtained for up to 30 mm from the screw insertion part (Fig 2B and 2C). The threshold was unified at −325.0 a.u. based on the assessment of the lesions of “air”, with the consensus of 2 spinal surgeons who had a special interest on implant study (D.N. and T.N. with 10 and 25 years of experience, respectively). The screw insertion areas were defined as the location of “air” surrounded by the 5.5-mm circles (outer diameter of the screw) (Fig 2D–2F). The average screw insertion area was used for analysis (Fig 2G). The imaging values were measured using the software ImageJ 1.48v (available at rsbweb.nih.gov/ij/).

### Insertion torque measurement

A digital torque gauge HTGA-5N (IMADA Co., LTD, Aichi, Japan) was used to measure the insertion torque (peak torque [36, 37]) at 40-mm insertion. The specifications of this torque gauge were as follows: accuracy ± 0.5% full scale ±1digit and sampling rate 2000 data/sec. The insertion torque (Nm) was measured as the screw was advanced into the material. The insertion torque was found to increase progressively, because the number of screw threads cutting through the bone increased as the screw advanced. Thus, the torque in the state of being inserted to an appropriate length was the maximum torque. Generally, the surgical operator felt this maximum torque as the fixation strength of the screw [36]. This torque was defined as the peak torque.

### Resonance frequency analysis

RFA was conducted using a specific device (Osstell ISQ, Osstell, Columbia, MD) without contacting the screw after pedicle screw insertion was completed. The materials were not held by a fixture during measurement and were placed on a normal laboratory table instead. The ISQ value was obtained from the Osstell ISQ® and ranged from 0 to 100, depending on the resonance frequency (Hz) of the pedicle screws. The higher the ISQ value, the more stable the implant [38]. The pedicle screws were vibrated via a micromagnetic wave, which generated inertial forces due to the magnets’ masses in a plane perpendicular to the axis of the screw. The size of the magnets was scaled according to the relative size of the pedicle screw with respect to a dental implant.

### Pull-out force measurement

**Fig 3:**
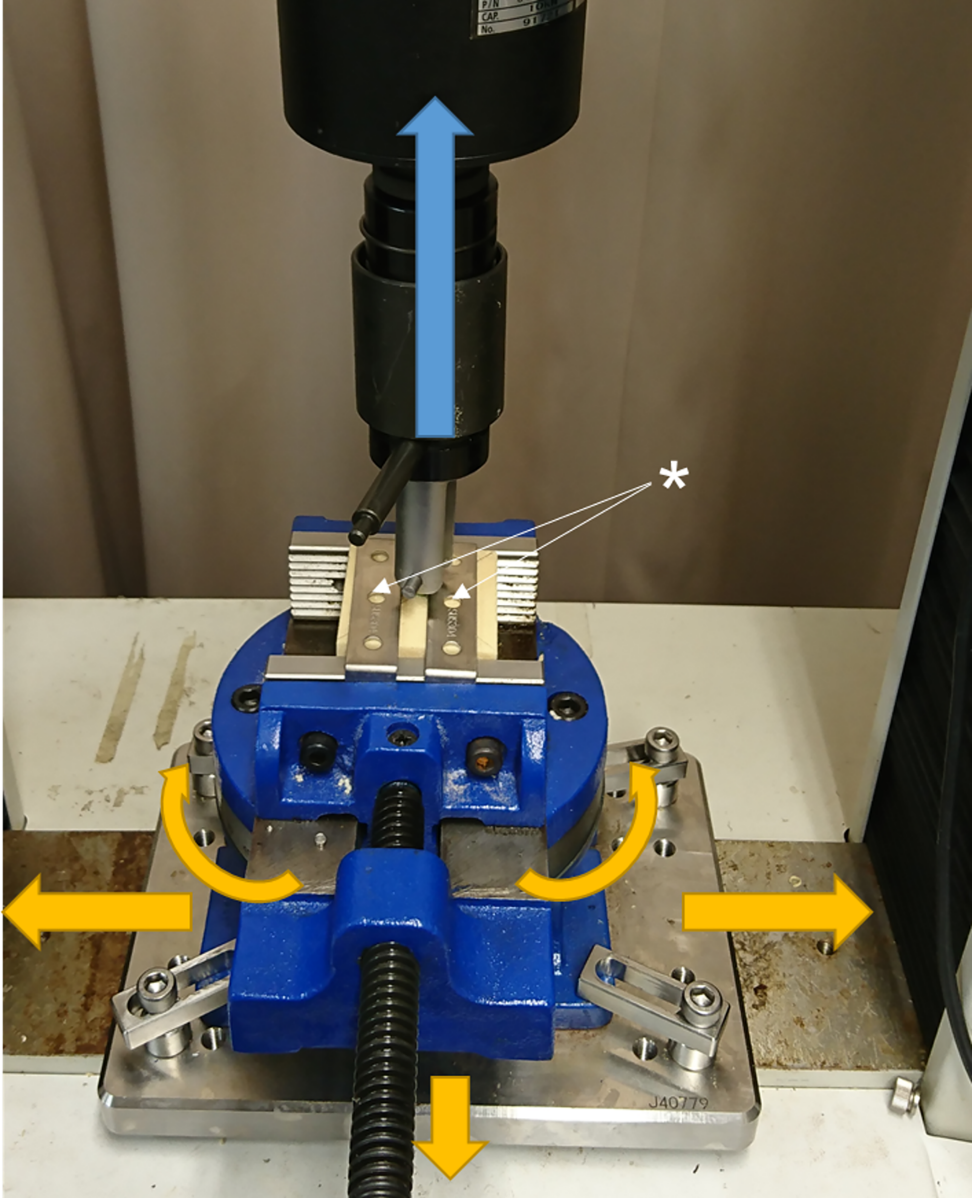
Pull-out force test. The material is pulled on a specially designed fixture without holding it from the sides. The base plate moves freely and combines the vice, which has a freely determined angle. It is structured to support on the upper surface of the material with plates (*) without clamping the material directly with the vice.

**Fig 4:**
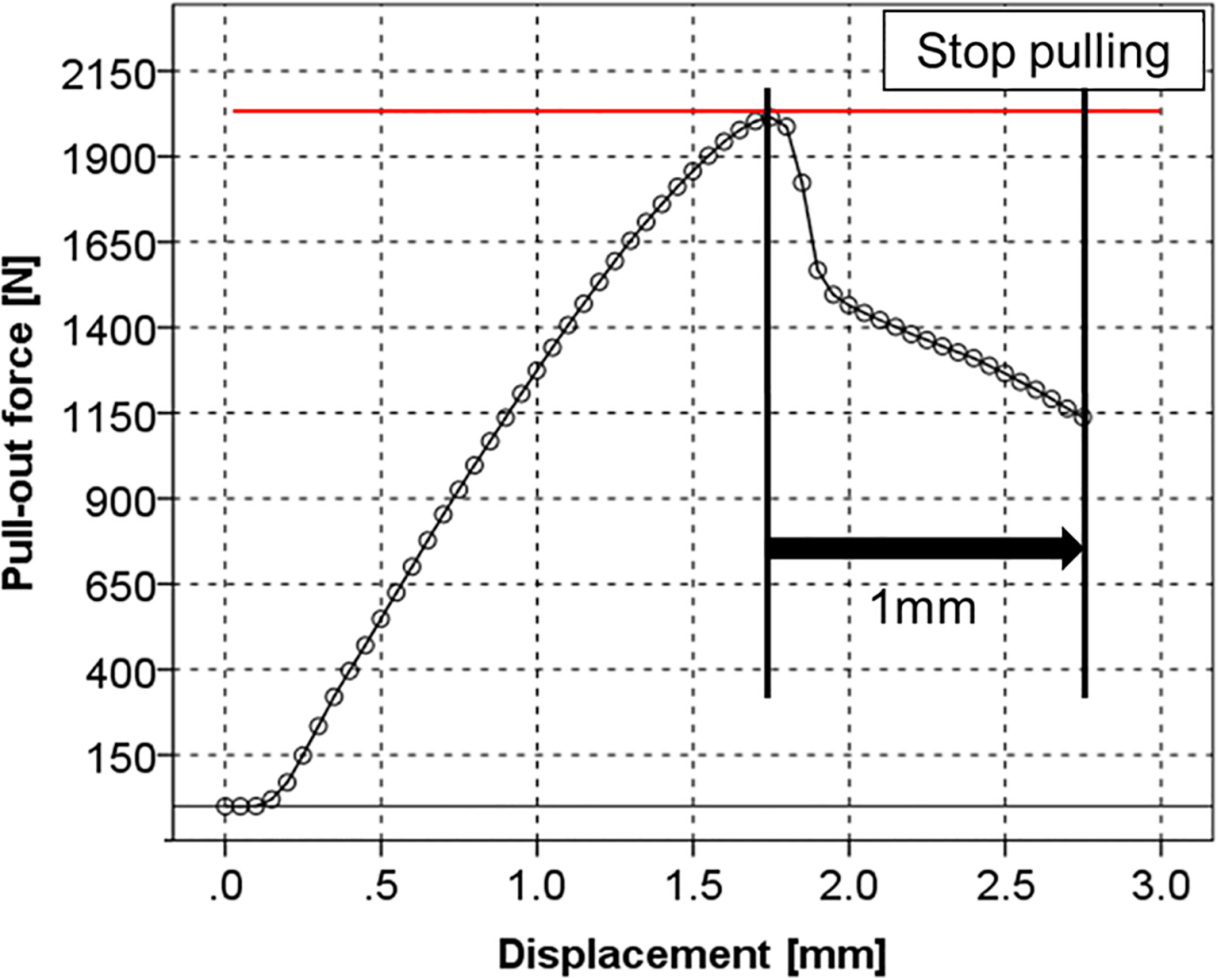
Load-displacement curve for the pedicle screw. The circles represent the actual data points. At 1 mm after the failure point (red line), pulling is stopped.

Pull-out force measurements were performed according to the ASTM-F543-07 testing standards [39]. The materials were placed on a specially designed fixture with a self-aligning function to keep a vertical pull-out alignment (Fig 3). The AG-IS 10kN (Shimadzu Corporation, Kyoto, Japan) was used to measure the maximum pull-out force at a testing speed of 5 mm/min [39]. The strength was continuously recorded in 0.1-mm increments until 1 mm after the failure point (i.e., the peak point in the load-displacement curve (Fig 4). At 1 mm after the failure point, pulling was stopped (Fig 4). We loaded the screw until 1 mm past the failure point because it was technically difficult to reproduce finishing the test exactly at the failure point and because examining the effect of loosening was one of the purposes of this study. The pedicle screws in the materials were removed carefully to preserve the structures of the materials. The free fragments in the insertion hole were removed using an air duster.

**Fig 5:**
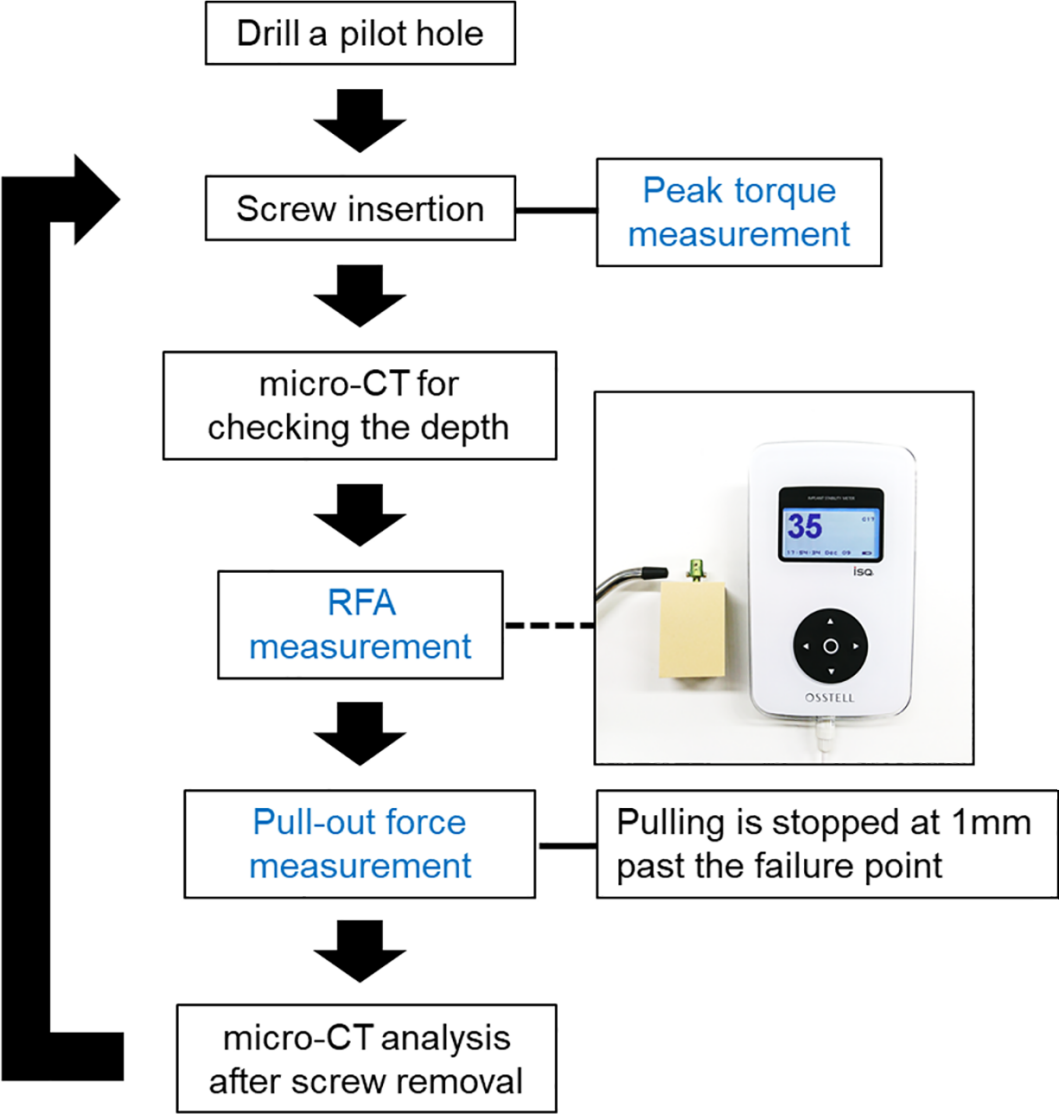
Flowchart of experiments.

The same tests (Insertion torque measurement, RFA, and pull-out force measurement) were repeated at the same insertion path 10 times to intentionally create loosening (Fig 5).

### Data analysis

The measurements of the 3 fixation strengths (peak torque, pull-out force, and the ISQ value) and the average screw insertion areas were collected from the 5 biomechanical test materials. These values were acquired in 10 steps for each biomechanical test material, because the screw insertion experiments were repeated 10 times in the manner described above (Fig 5).

Statistical calculations were performed using SPSS statistics software version 24 (International Business Machines Corporation, Armonk, NY, USA). Box-and-whisker plots of the 3 fixation strength measures and the average screw insertion areas were drawn every time the insertions were repeated. One-way analysis of variance with post-hoc analyses for repeated measures were performed to evaluate the differences in the 3 fixation strength measures and the average screw insertion areas among the number of screw insertions.

A simple regression model and a two-piece linear regression model with minimized sums of the squared residuals of the 2 types of regression were used to analyze the relationships among the 3 fixation strength measures and the average screw insertion areas. To assess the goodness of fit of these regression models, Akaike’s information criterion (AIC) was used [40, 41]. When the AIC difference between the two models was greater than 1, the model with the smaller AIC was considered to have a significantly better fit [42]. The significance level of all tests was set at p = 0.05.

## Results

**Fig 6:**
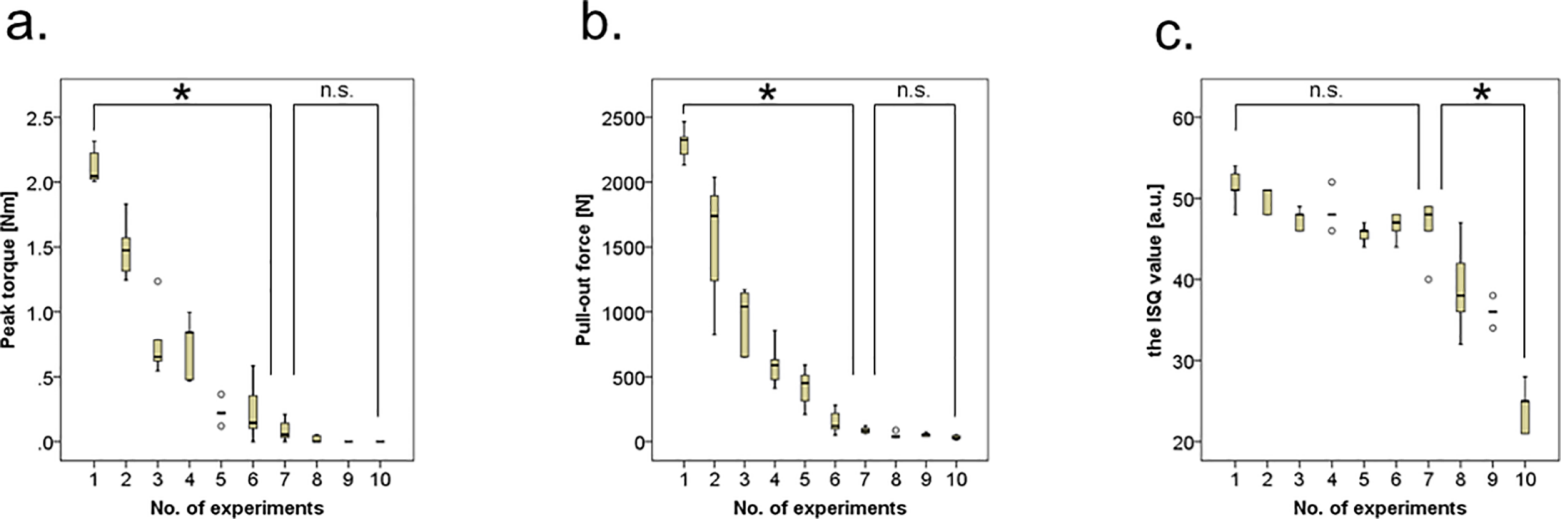
Results of 3 fixation strength measures for each insertion number. Box-and-whisker plots: the bottom and top of the box are the first and third quartiles; whereas the band inside the box is the second quartile (i.e., median). The ends of the whiskers represent the minimum and maximum data, excluding the outliers (white circles). One-way analysis of variance with post-hoc analyses was performed to evaluate the differences in the 3 fixation strength measures among the number of insertions. Peak torque (a), pull-out force (b), and the ISQ value (c). n.s.: not significant; *p <0.05.

Fig 6 shows the results of the 3 fixation strength measures for each insertion number. The peak torque and pull-out force decreased significantly with repeated screw insertion and plateaued after 7 repetitions (Fig 6A and 6B). On the other hand, the ISQ value did not decrease significantly with less than 7 screw insertions, but it decreased significantly after exceeding 7 times of screw insertion (Fig 6C).

**Fig 7:**
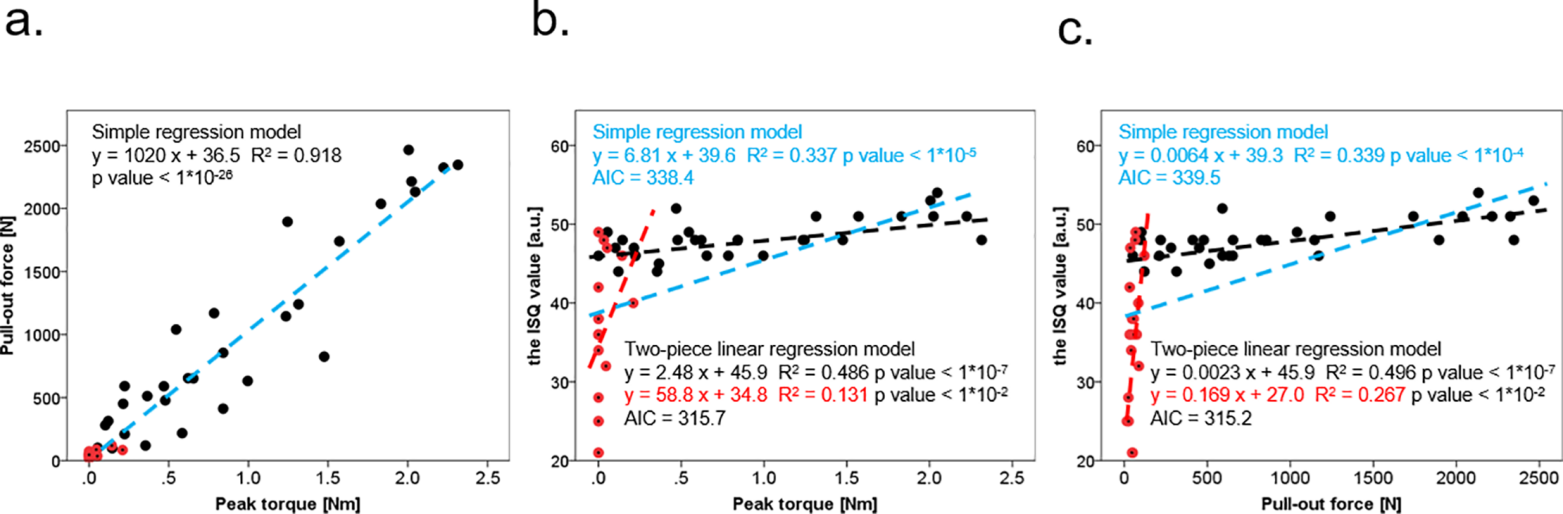
Simple and two-piece linear regression analyses of the relationships among the 3 fixation strength measures. Scatter plots and best fit lines of linear regression between peak torque vs. pull-out force (a), peak torque vs. the ISQ value (b), pull-out force vs. the ISQ value (c). Black dots: the results of screw insertions 1 to 6; red dots: the results of screw insertions 7 to 10; blue dotted line: best fit line of the results from screw insertions 1 to 10; black dotted line: best fit line of the results from screw insertions 1 to 6; red dotted line: best fit line of the results from screw insertions 7 to 10. AIC: Akaike’s information criterion; R^2^: coefficient of determination.

Fig 7 shows the relationships among the 3 fixation strength measures. There was a strong correlation between peak torque and pull-out force (R^2^ = 0.918, p < 1×10^-26^) (Fig 7A) in the simple regression model. However, the relationships of the ISQ value with the peak torque (R^2^ = 0.337, p <1×10^-5^) and the pull-out force (R^2^ = 0.339, p < 1×10^-4^) were not as strong as the relationship between peak torque and pull-out force (Fig 7B and 7C) in the same model. Interestingly, a 2-piece linear regression analysis showed that after 7 screw insertions, the ISQ value decreased sharply, compared with the peak torque (simple regression model: AIC = 338.4, two-piece linear regression model: AIC = 315.7) and pull-out force (simple regression model: AIC = 339.5, two-piece linear regression model: AIC = 315.2).

**Fig 8:**
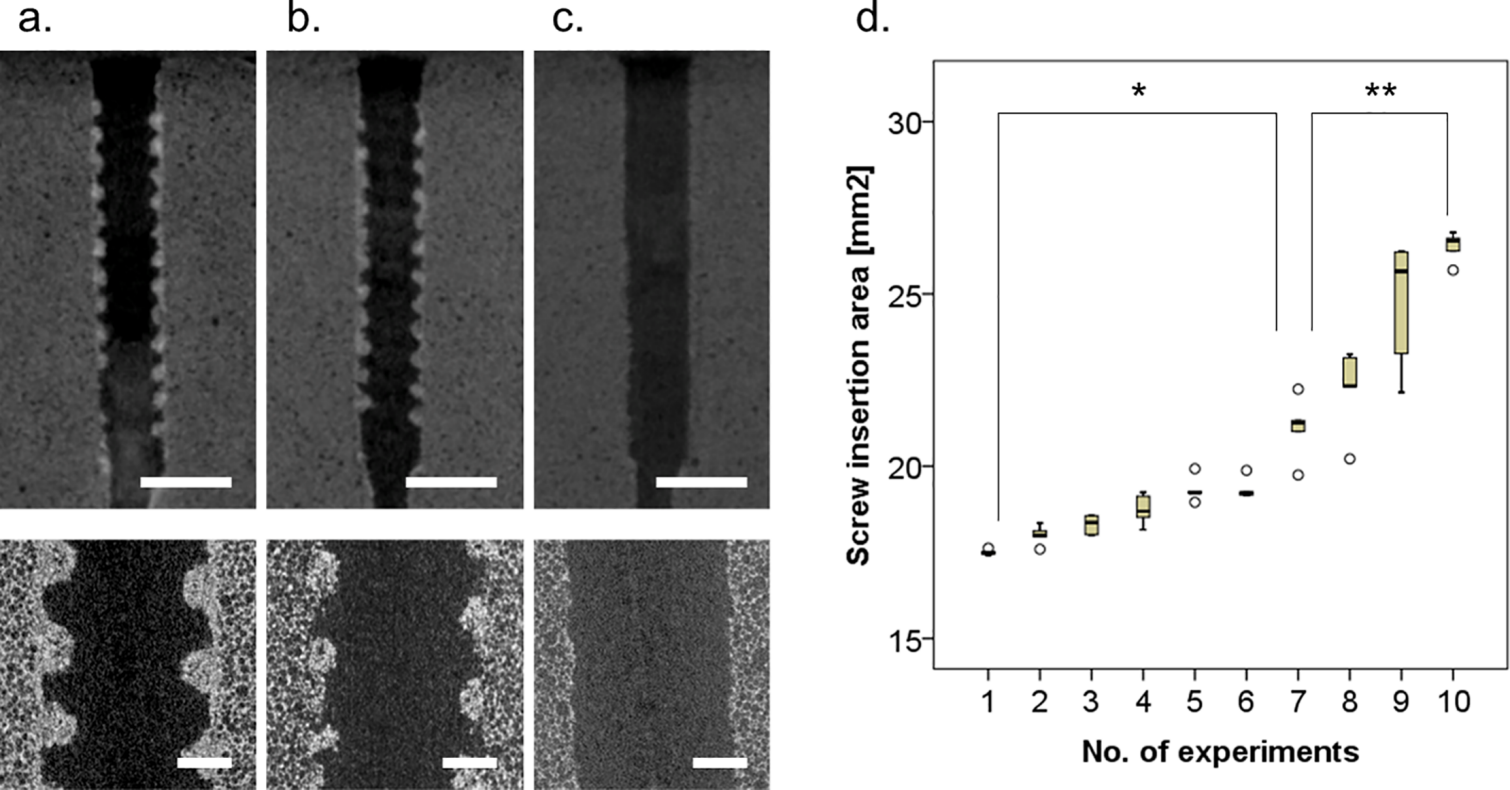
**Micro-CT sagittal image of the biomechanical test materials after screw insertions and removal 1 time (a), 7 times (b), and 10 times (c). (d) The results of screw insertion area for each number of insertions.** (a,b,c) top: low-power field, scale bar: 10 mm; bottom: high-power field, scale bar: 2 mm. (d) *p <0.05, ** p <0.01.

To confirm the structural changes in the biomechanical test materials, micro-CT image analysis was performed (Fig 8). Even after 7 insertions, the major and minor diameters of the internal threads seemed to be preserved in the low-power field. On the other hand, in the high-power field, the volume of the major and minor diameters of the internal threads seemed to be maintained, as shown in the low-power field, but their shape became rounded with repeated screw insertions. As a result, the material integrity of the internal threads appeared destroyed (Fig 8B). With 10 screw insertions, the major and minor diameters of the internal threads disappeared (Fig 8C). Fig 8D shows quantification of the screw insertion area. After 7 screw insertions, the screw insertion area increased more sharply along with the decrease in the material volume of the major and minor diameters of the internal threads.

**Fig 9:**
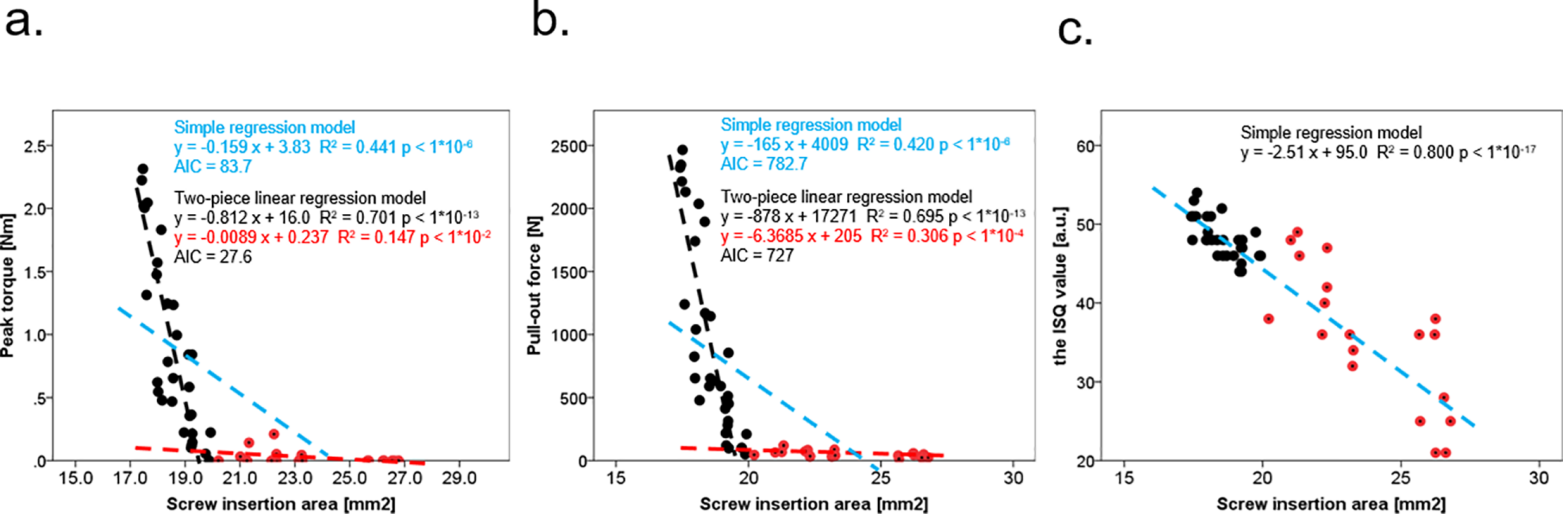
Simple and two-piece linear regression analyses of the relationships between screw insertion area and the 3 fixation strength measures. Scatter plots and best fit lines of linear regression between screw insertion area vs. peak torque (a), screw insertion area vs. pull-out force (b), and screw insertion area vs. the ISQ value (c). Black dots: the results from 1 to 6 screw insertions; red dots: the results from 7 to 10 screw insertions; blue dotted line: best fit line of the results from 1 to 10 screw insertions; black dotted line: best fit line of the results from 1 to 6 screw insertions; red dotted line: best fit line of the results from 7 to 10 screw insertions. AIC: Akaike’s information criterion; R^2^: coefficient of determination.

Fig 9 shows the relationships of the screw insertion area with the 3 fixation strength measures. Two-piece linear regression analysis showed that peak torque and pull-out force decreased sharply before 7 insertions (Fig 9A and 9B). That is, even if the material volume between the major and minor diameters was maintained, peak torque and pull-out force decreased if the material integrity between the major and minor diameters was destroyed. On the other hand, the ISQ value declined simply due to the destruction of the material volume between the major and minor diameters and the resulting increase in screw insertion area (Fig 9C).

## Discussion

Several studies have evaluated the stability of orthopedic implants using insertion torque and pull-out force [8, 13, 43–49]. However, these techniques of fixation strength measurement are invasive and non-repeatable. In the present study, the ISQ value, a non-invasive and repeatable measurement technique, was used for the evaluation of pedicle screw stability. RFA with the Osstell® has been used to evaluate dental implant stability [28].

**Fig 10:**
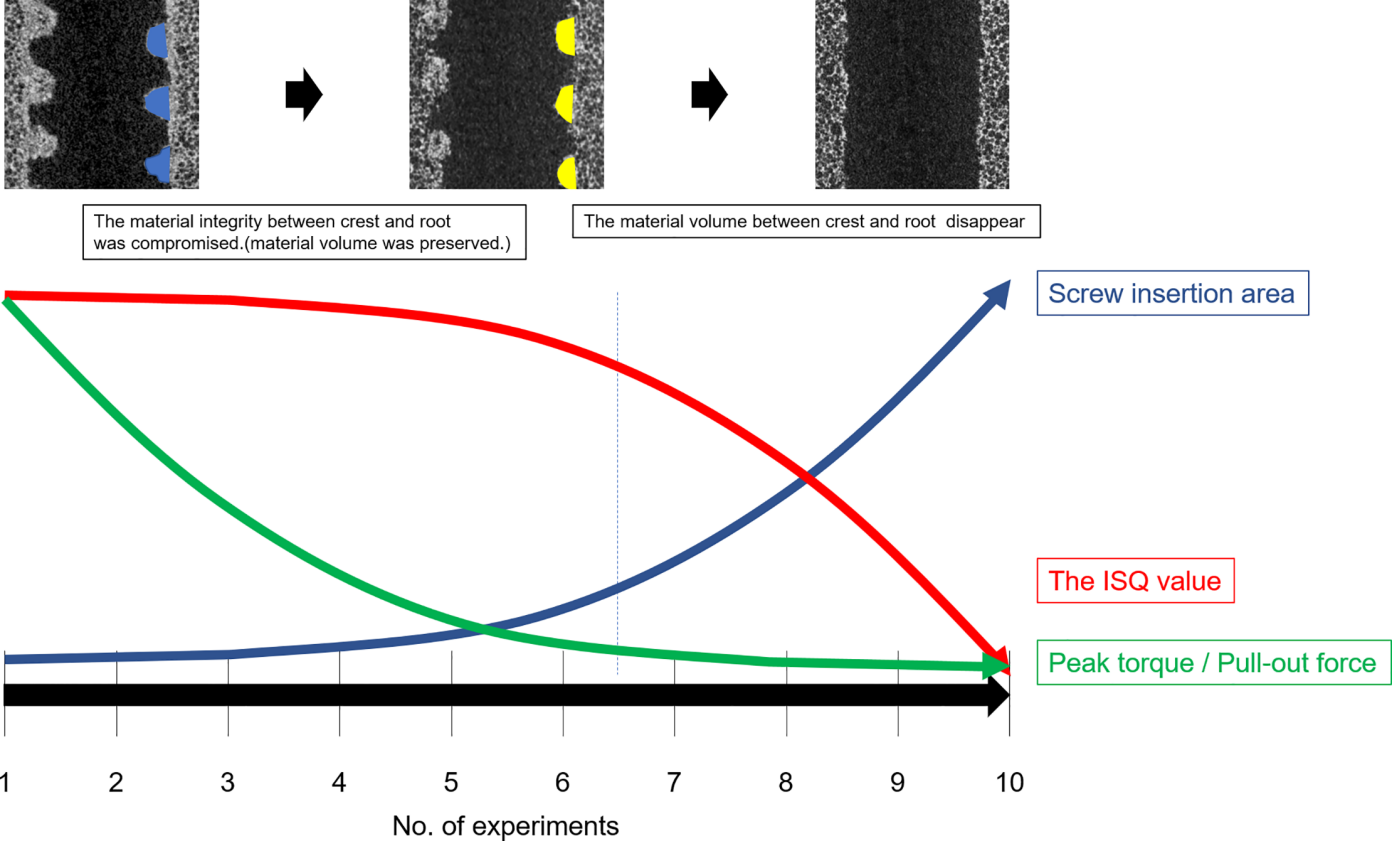
A qualitative schema of the relationships among the 3 fixation strength measures, screw insertion area, and the number of screw insertions.

Fig 10 showed a schema of the relationships among the 3 fixation strength measures, screw insertion area, and the number of screw insertions.

With repeated screw insertion, the material integrity between the major and minor diameters of the internal thread is first compromised, but the material volume is preserved; however, at the end, material volume also disappears. Considering these mechanisms, peak torque and pull-out forces decrease along with the destruction of material integrity, whereas the ISQ value decreases along with the destruction of material volume that occurs after the destruction of material integrity. There was a strong relationship between peak torque and pull-out force, which suggested that the 2 were similar fixation strength measures. Meanwhile, the weak relationship of the ISQ value with both peak torque and pull-out force in the simple regression model was likely caused by the fact that the ISQ value was retained more than peak torque and pull-out force, even with repeated screw insertion and removal.

In the present study, loosening was created artificially to perform pull-out force measurement and to repeat screw insertion and removal. All fixation strength measures decreased with loosening. Sanden et al reported in an animal study that in cases with pedicle screw loosening on X-ray images, the bone structure between the major and minor diameters was destroyed, and that the screw insertion areas were scaled to a cylindrical shape histologically [50]. In the present study, the structural changes in the environment surrounding the implant until a completely loosened state was simulated in the biomechanical test materials with 10 insertions, and the changes in each fixation strength measure were followed. With repeated screw insertion and removal, the material volume between the major and minor diameters disappeared after the material integrity was lost. Therefore, the peak torque and pull-out force decreased only after the destruction of material integrity, whereas the ISQ value was retained and correlated with the screw insertion area.

Pull-out force is the fixation strength measure that reflects axial load [15] and is determined by the resistance of the minute material structure between the crest and the root of the screw. Upon pull-out force measurement and repeated screw insertions to create loosening, the material integrity between the crest and the root is easily destroyed, because this area is small enough to collapse by simply repeating screw insertions. For this reason, the pull-out force and its similar fixation strength measure, the peak torque, decreased easily only with destruction of material integrity.

In the dentistry field, the ISQ value has been reported to correlate negatively with the displacement of an implant after application of a lateral load [28, 30]. This suggested that the ISQ value reflected the screw stability against loading in the tangential plane, i.e., screw toggle. In other words, the ISQ value reflects the ability of the screw to withstand the force pushing the screw sideways on the external screw surface, not the external screw thread structure. Thus, the ISQ value may be related to the resistance of the broader material structure beyond the major and minor diameters of the internal thread. Therefore, the ISQ value did not decrease with breakdown of the material structure between the major and minor diameters of the internal thread; it only decreased after disappearance of the material volume between the major and minor diameters of the internal thread, which was the result of more destruction.

In vivo, stress force against the implant is applied not only in the axial direction, but also in various directions [51]. Therefore, the conventional fixation strength measures, insertion torque and pull-out force, do not necessarily reflect the stress force *in vivo* [51]. In the present study, the ISQ value, which represents a multidirectional fixation strength [31], was shown to be a type of index that had characteristics that were different from those of the conventional fixation strength measures.

This study had some limitations. First, the Osstell ISQ® is a system for evaluating implant stability in the field of dentistry, and this method for measuring resonance frequency is not established in the field of orthopedics. Modification of the RFA system to make it suitable for orthopedic implants is needed. Since the Osstell ISQ® is one of the most popular devices used to evaluate implant stability in the dental field [52] and it is easy to handle during stability measurement, we applied the Osstell ISQ® in our experiment. Second, biomechanical test materials were used as substitute for human bone. There were some differences between the mechanical characteristics of these materials and those of actual vertebrae. For clinical application, these results should be used with caution. Further cadaveric and in-vivo studies are warranted before evaluating the feasibility of this technique for spine surgery. Third, the influence on the fixation strengths of the cortical bone structure peculiar to the vertebral body was not considered. Fourth, given the problem of metal artifacts on micro-CT images, the structure surrounding the pedicle screw was examined with the screws removed. However, these results can be considered a preliminary foundation to explain the characteristics of the ISQ value, relative to the other fixation strength measures and the structure of the tissue surrounding the implants. Finally, all of the specimens were prepared and examined in a uniform and reproducible manner. We believe that this study provided a new perspective on the definition of implant fixation strength and presented useful information on implant design development.

## Conclusion

The ISQ value reflects the fixation strength against the perpendicular direction, unlike conventional fixation strength measures.
